# Design Considerations for an Exergame-Based Training Intervention for Older Adults With Mild Neurocognitive Disorder: Qualitative Study Including Focus Groups With Experts and Health Care Professionals and Individual Semistructured In-depth Patient Interviews

**DOI:** 10.2196/37616

**Published:** 2023-01-05

**Authors:** Patrick Manser, Manuela Adcock-Omlin, Eling D de Bruin

**Affiliations:** 1 Motor Control and Learning Group – Institute of Human Movement Sciences and Sport, Department of Health Sciences and Technology, ETH Zurich Zurich Switzerland; 2 Division of Physiotherapy, Department of Neurobiology, Care Sciences and Society, Karolinska Institutet Stockholm Sweden; 3 Department of Health, OST – Eastern Swiss University of Applied Sciences St.Gallen Switzerland

**Keywords:** cognition, exercise, exergame, design, development, neurosciences, technology, training

## Abstract

**Background:**

Exergames have attracted growing interest in the prevention and treatment of neurocognitive disorders. The most effective exergame and training components (ie, exercise and training variables such as frequency, intensity, duration, or volume of training and type and content of specific exergame scenarios) however remain to be established for older adults with mild neurocognitive disorders (mNCDs). Regarding the design and development of novel exergame-based training concepts, it seems of crucial importance to explicitly include the intended users’ perspective by adopting an interactive and participatory design that includes end users throughout different iterative cycles of development.

**Objective:**

This study aimed to determine the capabilities, treatment preferences, and motivators for the training of older adults with mNCD and the perspectives of individuals on training goals and settings and requirements for exergame and training components.

**Methods:**

A qualitative study including expert focus groups and individual semistructured in-depth patient interviews was conducted. Data were transcribed to a written format to perform qualitative content analysis using QCAmap software.

**Results:**

In total, 10 experts and health care professionals (80% females) and 8 older adults with mNCD (38% females; mean age 82.4, SD 6.2 years) were recruited until data saturation was observed.

**Conclusions:**

The psychosocial consequences of patients’ self-perceived cognitive deterioration might be more burdensome than the cognitive changes themselves. Older adults with mNCD prefer integrative forms of training (such as exergaming) and are primarily motivated by enjoyment or fun in exercising and the effectiveness of the training. Putting the synthesized perspectives of training goals, settings, and requirements for exergames and training components into context, our considerations point to opportunities for improvement in research and rehabilitation, either by adapting existing exergames to patients with mNCDs or by developing novel exergames and exergame-based training concepts specifically tailored to meet patient requirements and needs.

## Introduction

### Background

The normal aging process is associated with a decline in physical and cognitive abilities [1,2]. When the cognitive decline exceeds the normal age-related cognitive decline but is not severe enough to interfere with independence in activities of daily living, it can be classified as “mild cognitive impairment” (MCI), representing an intermediate stage of cognitive impairment between the normal aging process and dementia [3-9]. The condition MCI has evolved over the last decades [5] and has recently been incorporated in the latest Diagnostic and Statistical Manual of Mental Disorders 5th Edition (DSM-5) and the International Classification of Diseases 11th Revision, referred to as mild neurocognitive disorder (mNCD) [7,9-11]. The prevalence of mNCD increases with age, while the incidence of mNCD and the progression to dementia is expected to rise, largely because of the globally growing life expectancies and sedentary lifestyles [3,5,12-15]. As currently no effective pharmacological interventions for patients with mNCD exist [16], alternative options to prevent and treat neurocognitive disorders are needed. Targeting modifiable risk factors in midlife may hold promise for mitigating or even preventing neurocognitive disorders in later life [17-21]. The modifiable risk factors for mNCD include the presence of vascular risk factors (ie, metabolic syndrome, hypertension, hyperlipidemia, coronary heart disease, diabetes mellitus, or stroke) [22-24] or a physically or cognitively sedentary lifestyle [25,26]. Consequently, changes in lifestyle that increase physical and cognitive activity and reduce vascular risk factors are protective against cognitive decline [27-35].

Exergames have gained growing interest to prevent and treat neurocognitive disorders [36-38]. “Exergaming is defined as technology-driven physical activities, such as video game play, that requires participants to be physically active or exercise in order to play the game” [39]. One of the major advantages of exergame-based training is that it is widely accepted by individuals with neurocognitive disorders. In addition, it increases training adherence and engagement by facilitating training motivation and satisfaction [40], which in turn may have a positive effect on the effectiveness of improving cognitive functioning [41]. Furthermore, exergames can be used as a form of simultaneous cognitive-motor training with incorporated cognitive task demands [42]. Meta-analytic evidence suggests that simultaneous motor-cognitive training is the most effective type of training for improving cognition in healthy older adults (HOA) [43,44] and older adults with mNCD [44-46]. For exergames specifically, a recent systematic review synthesized evidence from low risk of bias studies showing that there were consistent positive effects favoring exergaming in people with mNCD and dementia [40]. Nonetheless, it is currently difficult to draw reliable conclusions about the effectiveness of exergaming in preventing and treating neurocognitive disorders because of the substantial variations in the exergame-based training used. Therefore, further investigations are needed for the establishment of effective exergame and training components (ie, exercise and training variables such as the frequency, intensity, duration, or volume of training and the type and content of specific exergame scenarios) for cognitive functioning that can be applied with confidence in evidence-based exergame interventions [36].

Regarding the design and development of novel exergames, it seems crucial to explicitly include the intended users’ perspectives [47]. Taking the characteristics, needs, and experiences into account should ensure adequate use and therefore the effectiveness of the solution. Baquero et al [48] pointed out that an end user–centered methodological design is most often adopted in the development of computer-based training programs for cognitive rehabilitation of older adults with neurocognitive disorders (NCDs). In an ideal case, this process fulfills the international standards for the development of programs including (1) understanding and specifying the context of use (type, characteristics and tasks of users, and physical or social environment), (2) specifying the user requirements, (3) producing design solutions, and (4) evaluating the design [48,49]. So far, only half of the studies reporting computer-based interventions took the standard “specification of user requirements” into account [48]. This has led to the recommendation that future studies in this field should use an interactive and participatory design that explicitly includes end users throughout different iterative cycles of development [48]. In short, it is important to systematically and thoroughly investigate the specific user requirements and preferences for an exergame-based training concept before it is designed and developed.

### Objectives

This study aimed to determine the capabilities, treatment preferences, and motivators for the training of older adults with mNCD and the perspectives of individuals on training goals and settings and requirements for exergame and training components.

## Methods

### Overview

This study is part of the national project “Brain-IT,” which began in August 2020 in Switzerland. The aims of the overall project are (1) to determine the most suitable components for exergame-based training in older adults with mNCD; (2) to explore novel strategies for a real-time adaptive exergame system to individually tailor exergame demands according to users’ physical or cognitive capabilities; (3) to incorporate the acquired knowledge into an exergame-based training concept with the aim of halting or reducing cognitive decline and improving quality of life; and (4) to evaluate the effectiveness of the resulting training intervention in older adults with mNCD. The project is guided by a theoretical framework that provides specific guidance in the design, development, and evaluation of exergames for older adults, the “Multidisciplinary Iterative Design of Exergames (MIDE): A Framework for Supporting the Design, Development, and Evaluation of Exergames for Health” [50], which provides specific guidance in the design, development, and evaluation of exergames for older adults. This study is part of the first phase of the project, with the aim to specify a *“*set of design requirements that includes design considerations, accessibility recommendations, user modeling elements, and technological reflections to be followed in the design and development phase*”* [47,50], and it was combined with an extensive literature review and reflections on technology scoping and sustainability strategy (see steps 4 and 5 of phase 1 of our recently published methodological paper [47]). For the project, the exergame device “Senso (Flex)” (Dividat AG) was preselected on the basis of (1) our previous research, (2) because this device has already been shown to be feasible and well-accepted in geriatric patients [51] and patients with major neurocognitive disorder [52], and (3) because it is already widely used (and therefore available more widely and for longer term by end users and health care institutions) for motor-cognitive training within geriatric populations, physiotherapies, or rehabilitation clinics in Switzerland. On this basis, this qualitative study was designed to achieve the defined objectives in general; in addition, it also aimed to collect evidence about the previous experiences of experts or health care professionals with different exergame systems [including the “Senso (Flex)”]. In this way, the project team wanted to collect evidence to make an informed decision whether the specific exergame device was suitable for the project, what possible modifications might be needed to optimize the exergame experience for patients with mNCD, and whether and what alternative exergame devices are suggested by the experts (see subsections of “(T2) Treatment Experience and Preferences—Previous Experiences with Exergames (‘Senso’ specifically)” and “(T5) Exergame and Training Components—Exergame System and Content” in the focus group discussions). Other than parts of these 2 sections that include device-specific findings, none of the remaining sections in this manuscript are device specific.

### Study Design

A qualitative study was conducted between November 2020 and January 2021, including expert focus groups and patient interviews; both were organized as semistructured, in-depth interviews. Semistructured, in-depth interviews are the most widely used interviewing format for qualitative research and are generally organized around a set of predetermined open-ended questions, with additional questions and discussion points emerging from the dialogue [53]. The study was planned and reported in accordance with the “consolidated criteria for reporting qualitative research (COREQ)” [54].

The MIDE Framework [50] guided our approach. On the basis of this framework, we integrated multiple stakeholders into the design and development process including exergaming researchers, clinical experts with different backgrounds, a company representing the exergaming industry, and the end users.

### Ethics Approval

All the study procedures were performed in accordance with the Declaration of Helsinki. The study protocol (not registered) was approved by the ETH Zürich Ethics Commission (EK 2020-N-154). All interested individuals were fully informed of the study procedures. The expected benefits and risks of the study were explained by the study investigator, who was also able to answer open questions and clarify individuals’ uncertainties. It was further verified that withdrawal was permitted at any time during the study without providing any reason. After sufficient time, suitable individuals willing to participate in the study provided written informed consent and were included in the study. No compensation was provided to the participants.

### Participants

#### Experts

Recruitment aimed at including experts and health care professionals experienced with exergame training of older adults with mNCD, preferably (but not necessarily) with the exergame training system “Senso (Flex)” or similar. For this purpose, Dividat AG was asked to provide a contact list of 10 to 15 external experts and health care professionals with a variety in age, sex, educational level, and experience in therapy of older adults with mNCD, who are not employed by Dividat AG or had received any funds from Dividat AG for their work. All recommended experts and health care professionals were contacted via email between November and December 2020. By applying broad inclusion criteria, a rich spectrum of experts and health care professionals were considered in the study, which in turn will foster the usability of the resulting program in clinical practice. The specific eligibility criteria comprised the following aspects: (1) experts or health care professionals (eg, physical therapists, movement therapists, neuropsychologists, or researchers experienced with exergames) experienced with exergame training or with older adults with mNCD; (2) German or English speaking; and (3) age ≥18 years. There were no specific exclusion criteria.

#### Older Adults With mNCD

Older adults with mNCD were consecutively recruited between November 2020 and January 2021 in collaboration with health care institutions and (memory) clinics in the larger area of Zürich. Leaflets and study information sheets containing researchers’ contact details were handed out to suitable patients by their therapists. Suitable patients were identified from medical records and patient registries of memory clinics or from diagnostics that had just been performed. Interested patients were contacted by the research team by telephone or email to clarify or obtain further information about the study procedures and to register interest in participating in the study. Subsequently, all patients were fully informed about the study procedures in a face-to-face meeting at the patient’s homes. In addition, patients of interest were screened for eligibility. The eligibility criteria are presented in Textbox 1.

**Description of all eligibility criteria.**

- Inclusion criteria
  Participants fulfilling all the following inclusion criteria were eligible:
    - (1= mild neurocognitive disorder [mNCD]) clinical diagnosis of “mNCD” according to International Classification of Diseases 11th Revision [7] or Diagnostic and Statistical Manual of Mental Disorders 5th Edition [9]) OR (2=sMCI). Patients “screened for MCI” according to the following criteria: (1) informant (ie, health care professional)–based suspicion of mild cognitive impairment confirmed by (2) an objective screening of mild cognitive impairment based on the German version of the using the Quick Mild Cognitive Impairment Screen [55] with (b1) a recommended cutoff score for cognitive impairment (mild cognitive impairment or dementia) of <62/100 [56], while (b2) not falling below the cutoff score for dementia (ie, <45/100 [56])
    - German speaking
- Exclusion criteria
  The presence of any of the following criteria led to exclusion:
    - Presence of additional, clinically relevant (ie, acute or symptomatic) neurological disorders (ie, epilepsy, stroke, multiple sclerosis, Parkinson disease, brain tumors, or traumatic disorders of the nervous system)
    - Presence of any other unstable or uncontrolled diseases (eg, uncontrolled high blood pressure, progressing or terminal cancer, etc)

### Procedures and Data Collection

#### Expert Focus Groups

The expert focus groups were moderated by the first author (PM) into groups of up to 5 experts. The moderator was a male doctoral student with a master’s degree in Health Sciences and Technology (ETH Zürich, Switzerland), who was trained for qualitative research. Owing to the COVID-19 pandemic, all focus group sessions were held as web-based meetings in the form of Zoom sessions (Zoom Video Communications), took approximately 60 to 90 minutes to complete, and were audio recorded. Each session started with a short presentation of the background and overall aim of the project. Subsequently, the aim of this study was presented before starting the focus group discussions. The focus group discussions were organized as semistructured, in-depth interviews with open-ended questions to enable open conversations [53]. The exchange was conducted following a focus group guide (Multimedia Appendix 1) structured along 5 topics, each consisting of multiple key questions. First, the capabilities of older adults with mNCD were discussed, in continuation with insights into training goals and outcomes in the perspective of patients as well as therapists. Thereafter, the exchange focused on treatment experiences and preferences as well as motivators for training of older adults with mNCD. Finally, the requirements and optimal components of the exergame-based training were critically discussed. To focus the moderator’s attention on participants’ verbal and nonverbal communication and because handwritten notes during interviews are considered relatively unreliable, no notes were taken during the focus group sessions [57].

#### Patient Interviews With Older Adults With mNCD

The patient interviews were conducted individually with each patient by the first author (PM) and either took place at ETH Zürich (Institute of Human Movement Sciences and Sport, Leopold-Ruzicka-Weg 4, 8093 Zürich) or at the patients’ homes, depending on the patients’ preferences. The interview sessions were held face-to-face in a quiet room with no one present besides the interviewer, the patient, and, if requested, a care professional or partner as personal support for the patient. We did not set a time limit for the interviews but gave all participants enough time to share their views on the topics discussed. On average, each session took approximately 20 to 30 minutes to complete and was audio recorded. Before starting the interview, the background and overall aim of the project as well as the aim of this study were explained to each patient. The interviews were organized as semistructured, in-depth interviews along an interview guide (Multimedia Appendix 1) [53]. The interview guide was not pilot-tested, as it was developed by the first author (PM) in collaboration with the second author (MA), an experienced clinical neuropsychologist. After questioning the patients’ capabilities as well as their previous treatment or training experience and preferences, the interview continued with questions about motivators for training and the preferred components of exergame-based training. Open-ended questions were asked to enable an open conversation [53]. To focus the moderator’s attention on patients’ verbal and nonverbal communication, no notes were taken during the interviews [57]. Finally, the interviewer was prepared to tailor the interview questions and communication style to the patients’ capabilities, and in case of higher levels of impairment, to adopt strategies suggested to optimize communication with patients with NCDs [58,59].

### Sample Size

The intended sample size was set at approximately 5 to 10 experts for the focus group sessions and 5 to 10 older adults with mNCD for the patient interviews; however, study participants were consecutively included until data saturation was reached [60].

### Data Analysis

First, all audio files were transcribed in written format in Microsoft Word in pseudonymized form. The transcripts were not returned to the participants for corrections or comments. To explore the perspectives of patients and experts or health care professionals, a qualitative content analysis was performed according to Mayring et al [61,62] using QCAmap software [62-64]. The first step in the analysis involved repeated readings of the transcripts and listening to the original audio files to gain a better understanding of the conversation content. Second, the type of analysis (ie, category assignment procedure) was predefined for each of the research questions (ie, key questions of the interview guide). In case of an inductive category assignment procedure, a selection criterion and level of abstraction were defined for each of the research questions. For deductive category assignments, each research question was operationalized into categories, and a corresponding coding guideline (ie, category label, category definition, anchor example, and coding rules) was formulated. On the basis of this, all transcripts were coded line-by-line (ie, including a revision of the category system after a pilot loop). Subsequently, each resulting list of categories was grouped into main categories, and inter- and intra-agreement checks were performed. Finally, the results of each key question were analyzed along the structure (including predetermined themes and topics) of the interview guide that was created according to the guidelines of the MIDE Framework [50]. Thus, the results were structured and analyzed in 2 main themes and 5 topics. First, the section “user modeling” that included 3 topics: (T1) capabilities of older adults with mNCD, (T2) treatment experiences and preferences, and (T3) motivators for training. Second, “therapeutic needs,” including (T4) training goals and outcomes and (T5) exergame and training components. Within the topic “(T1) capabilities of older adults with mNCD,” the described cognitive capabilities and difficulties were classified into the key neurocognitive domains (as defined by Sachdev et al [10]) in line with DSM-V [9] on agreement between the first (PM) and second author (MA; an experienced neuropsychologist). Within the topic “(T3) motivators for training,” the motivators for training were coded and analyzed against the background of the “Self-determination Theory” [65]. The Self-determination Theory [65] accounts for the quality of different levels of motivational regulation in physical activity settings. It is considered useful to gain a better understanding and promote training motivation, enjoyment, and adherence and has demonstrated considerable efficacy in explaining exercise motivation and behavior [66-70]. Data from the qualitative content analysis were combined with quantitative data (ie, frequency of various statements [f] and in the case of patient interviews, the proportion of patients making a statement [in %]) [60]. The coding and data analysis process was cross-checked to enhance the credibility of the analytic procedure [60].

## Results

### Participants

In total, 11 external experts and health care professionals were contacted by the first author (PM). All experts responded and were interested in participating. Two experts could not participate in the focus group sessions because of time constraints. According to the “integrative” contribution of the MIDE Framework, *“*perspectives of various stakeholders (*e.g.*, industry partners, data analysts, health care professionals) are considered in the process of designing and developing exergames*”* [50]. In accordance with this, the founder of Dividat AG was involved in one of the focus group discussions as an industry representative. In total, 10 experts and health care professionals (80% females) participated in 1 of the 5 focus group sessions until data saturation was observed and further recruitment was terminated. The focus group sessions were conducted in groups of between 1 (*k*=3) and 3 (*k*=1) experts (median 1.5) and the moderator (PM). The professional backgrounds of the experts and health care professionals included exergaming researchers (n=4), physical and occupational therapists (n=2), neuropsychologists (n=2), project manager therapy (n=1), and founder of an exergaming company (n=1).

For the patient interviews, 8 patients (38% females; mean age 82.4, SD 6.2 years; mean level of cognitive functioning, measured by the German Version of the Quick Mild Cognitive Impairment Screen [55], 56.0, SD 8.2) were invited and interviewed until data saturation was observed and further recruitment was terminated. None of the patients refused to participate or dropped out of the study after providing their written informed consent. The demographic characteristics of the patients are summarized in Table 1.

**Table 1:** Demographic characteristics of the study population.

		Total sample (n=8)
Age (years), mean (SD)		82.4 (6.2)
BMI (kg/m^2^), mean (SD)		23.1 (2.4)
Physical activity (min/week), mean (SD)		298.8 (227.0)
Qmci^a^ [55] total score (points), mean (SD)		56.0 (8.2)
**Clinical subtype, n (%)**		
	mNCD^b^ due to Alzheimer disease	6 (75)
	Mild frontotemporal NCD^c^	0 (0)
	mNCD with Lewy bodies	0 (0)
	Mild vascular NCD	2 (25)

^a^Qmci: Quick Mild Cognitive Impairment Screen.

^b^mNCD: mild neurocognitive disorder.

^c^NCD: neurocognitive disorder.

### Qualitative Content Analysis Results

#### T1: Capabilities

The experts described a large variety of impairments observed in older adults with mNCD. The most frequently described impairments referred to cognitive functioning (f=43), including impairments in the following neurocognitive domains: executive function (f=23), complex attention (f=11), learning and memory (f=7), visuospatial skills (f=2), language (f=1), and social cognition (f=1). These cognitive changes were also described as affecting psychosocial factors (f=22), mainly by causing psychological distress (f=9) and feelings of insecurity (f=2), leading patients to try to hide their impairments from others (f=2). In addition, an increased fall risk (f=9) and reduced physical resilience (f=7) were observed. Although experiencing difficulties in activities of daily living (ADLs; f=1), patients were described as maintaining their functional independence in ADL (f=2).

In line with the experts’ viewpoint, cognitive deterioration (f=22, n=7, 88%) was frequently described by the patients, mainly affecting learning and memory (f=11, n=4, 50% of patients), executive function (f=6, n=4, 50% of patients), and complex attention (f=5, n=2, 25% of patients), whereas only minor restrictions in physical capabilities and mobility were mentioned (ie, impaired balance, [f=2, n=2, 25% of patients], reduced gait speed [f=1, n=1, 13% of patients], increased fall risk [f=9, n=5, 63% of patients], fatigue [f=6, n=3, 38% of patients], and joint pain [f=2, n=2, 25% of patients]). ADLs remained preserved in all patients, but the need for coping strategies was mentioned by 4 patients (50%) to be able to preserve ADLs. From the patients’ perspective, the consequences of their self-perceived subjective cognitive decline (f=8, n=6, 75% of patients) with regard to psychosocial factors were most frequently reported (f=36, n=8, 100% of patients), mainly involving psychological distress (f=13, n=2, 25%), feelings of insecurity (f=6, n=3, 38% of patients), depressive symptoms (f=2, n=2, 25% of patients), or fear of repeated falls (f=3, n=1, 13% of patients):

A really tedious thing is that you often can’t keep up. For example, in discussions or conversations. [...] You often think about what the other(s) have just said and in the meantime he or she has already continued. That’s why you often just don’t say anything. Of course, most people like it when you don’t say anything (*laughs*). So, these people don’t get upset about it. But I am.

P-01

I used to go running a lot. I don’t do that anymore. But swimming is still fine. In the worst case, I become a drowned corpse, but at least I can’t fall while swimming.

P-02

I can actually do everything; I just have to be careful because of my dizziness and weakness so that I don’t fall. I also have problems with short-term memory. I have to try to remember everything somehow, but I still forget a lot of things.

P-04

#### T2: Treatment Experience and Preferences

##### Previous Treatment and Training Experiences

To counteract cognitive decline and preserve physical capabilities, mobility, and ADLs, patients have already been on medical training therapy (MTT; f=3, n=3, 38% of patients), have already been on physical therapy (PT; f=2, n=2, 25% of patients), have performed a specific group-based (ie, f=1, n=1, 13% of patients) or individual (f=1, n=1, 13% of patients) cognitive training or meditation (f=1, n=1, 13% of patients), or have reported to have no experience in any specific therapy or training (f=1, n=1, 13% of patients).

From the patient’s viewpoint, MTT and PT were perceived as useful (f=3, n=3, 38% of patients), but patients reported that they would have to do it more consistently to profit from it (f=2, n=2, 25% of patients). Computerized cognitive training (CCT) was also perceived as useful (f=1, n=1, 13% of patients) and reported to be challenging, fun, and enjoyable (f=2, n=2, 25% of patients). Nonetheless, patients reported being insecure about the effectiveness of CCT (f=2, n=2, 25% of patients):

[In response to PT] [...]my gait has improved. I now take slow and long steps and no longer fall over. However, I would definitely have to do it more consistently.

P-02

The problem is primarily that my physical therapist only has time for me every 14 days because she is so booked up. Of course, it would be nice if I could go more often. But it is what it is, and I have to live with it.

P-08

[Patient explains game tasks of CCT] It’s not even that simple. This is all fun and useful. But I don’t know if it will do any good. [and] I have no intention of stopping. However, at some point I have to ask myself: “Does is go any further? Or is it just going to stay at what I’m currently able to manage?”

P-01

According to the experience of experts and health care professionals, only cognitive forms of training or physical exercises were often experienced as boring over time by older adults with mNCD (f=2) and required guidance by a therapist (f=2). More integrative forms of training, including gamified tasks close to everyday life, multimodal animation, and acoustic feedback, were reported to be preferred by patients (f=4):

It is often the case that patients are completely dependent during strength training, [and] [...]they just kept on exercising and exercising. [...] They often continue the exercises until you stop them.

E-10: founder of an exergaming company

Cognitive exercises including “a certain closeness to everyday life and also a multimodal animation[...] and acoustic feedback have been very well received.”

E-03: neuropsychologist

##### Previous Experiences With Novel Technologies

Although being described as skeptical about the use of technological devices, experts perceived older adults with mNCD as ready to use technological devices such as heart rate monitors during training (f=9), if its usability is ensured:

Well I think using a sensor it’s not a problem if the wearable is well designed.

E-01: exergaming researcher

Many people would certainly be okay with a Polar chest strap, but a monitor to be worn at the wrist would certainly be preferable. If people are told why these sensors are used and what they are measuring, it should be feasible with the chest sensors as well. It may be difficult with certain older ladies or overweight individuals, but for the average individual this should not be a problem.

E-03: neuropsychologist

The experts’ perceptions coincided with those of patients. All patients were willing to use a heart rate monitor worn with a chest strap during training, provided it was beneficial for their training. In addition, 75% (6/8) of patients stated that their PC or television was usable, whereas 25% (2/8) of patients reported limited usability:

[Regarding the use of heart rate monitors during training] [...]provided it’s useful I would be ready to wear such a heart rate monitor without having any reservations at all.

P-01

[About the usability of the television] Sure! All you have to do is press the switch. That’s still possible.

P-07

[About the usability of the personal computer] Yes, using my personal computer works more or less. [...] It is just not something of my generation. I have a computer and I use it, but there are always things I can’t do and have to ask my granddaughter.

P-01

##### Previous Experiences With Exergames [ “Senso (Flex)” Specifically]

None of the interviewed patients reported any previous experience with exergames in general or with the exergaming system “Senso (Flex)” specifically. Nonetheless, after a short introduction to the system, all patients stated that they would be willing to try it.

On the basis of the previous experiences of the experts and health care professionals, the interaction with the “Senso,” its overall usability, and the design of the exergames have been described as good (f=5). Regarding hardware components, minor usability problems have been reported. Patients were observed to unintentionally walk off the middle plate without noticing the feedback on the screen (f=4), constantly change their focus between the game tasks on the screen and the stepping plate to anticipate and plan their movements (f=4), or make too small steps to tap on one of the outer stepping plates (f=1). In addition, the patients needed time to familiarize themselves with the sensitivity of the stepping plate (f=2):

[...]the “Senso” is in general well usable and is also very often used.

E-04: exergaming researcher

The tasks on the “Senso” are very well designed.

E-08: project manager therapy

[...]the “Senso” is already very user friendly, [but] I had a little problem at the beginning of the experiment where people would accidentally go out of the square in the middle of the “Senso”.

E-09: exergaming researcher

Most of the time, the patients look down at that very moment and thus do not see the message [on the screen] at all.

E-07: physical and occupational therapist

Additional usability issues were reported to be linked to the capabilities of older adults with mNCD. First, it has been described that patients are often cognitively overloaded when trying out new games (f=1), by the occurrence of an unexpected situation or technical errors (f=2), or by the cognitive task demands required to interact with the exergame system in general (f=1), which may limit training duration owing to attentional exhaustion (f=2):

With new games, patients are often overwhelmed in general, because they don’t know what to expect. They often need time to find their way around.

E-07: physical and occupational therapist

[...] Patients are completely overwhelmed as soon as something unexpected or a technical problem occurs.

E-03: neuropsychologist

In contrast, the physical capabilities were reported to not directly affect the usability of the system (f=4), although some patients experienced difficulties with backward steps (f=2), and many patients made use of the handrail to reduce the physical strain (f=6). In some cases, physical limitations (eg, fatigue and joint pain) resulting from static loading have been reported to limit the training duration (f=4):

Patients often have problems with backward steps. [and] Patients hold on to the handrail far too often. [...] it is often the case that people hold on because it is simply ‘a bit more comfortable.

E-10: founder of an exergaming company

Often it is already difficult and tiring for patients to stand for a longer period of time. It is often easier for them to walk. [and] However, it should be noted that this form of fatigue is not necessarily comparable to fatigue caused by physical training. Fatigue does not necessarily come from physical exertion. It is possible that this type of fatigue is caused by the static load and the resulting joint pain.

E-06: physical and occupational therapist

When considering the specific games of the exergaming device “Senso” (video illustrations and explanations of all currently available games can be found at [71]), the simple and clear design structures of the games (f=4) and the intuitive tasks were reported to be highly appreciated by patients and promote good comprehensibility, which was reported for the games “Simple” (f=3), “Birds” (f=3). Nonetheless, there are also games that were reported to cause problems of understanding, in particular the games “Simon” (f=3), “Tetris” (f=3), “Habitats” (f=4), “Targets” (f=1), and “Snake” (f=2). These problems may be related to the game instructions (f=9):

[...]Many people are very happy with simple design structures. This should be maintained at all costs when designing new games for MCI patients. However, [...]some kind of adjustment of the game instructions is definitely needed.

E-10: founder of an exergaming company

For patients, a game does not stand out by its great graphics, but by the game tasks as such.

E-08: project manager therapy

[About problems of understanding the games] I think the reasons were that they didn’t really understand the instructions well.

E-09: exergaming researcher

However, it could also be related to the task demands of the games. It was reported that the patients need some time to familiarize themselves with the game to fully understand it (f=1). According to the experts’ experiences, this works well with the games “Simple” (f=4), “Birds” (f=1), “Flexi” (f=1), and in some cases “Habitats” (f=1). At the same time, games such as “Flexi” (f=1), “Habitats” (f=6), “Hexagon” (f=3), “Simon” (f=6), “Ski” (f=4), “Targets” (f=12), and “Tetris” (f=4) were frequently reported to start at an already (too) challenging level for older adults with mNCD and progress too fast while there is a limited range of games or adaptability of task demands at the lower end of difficulty levels (f=9). This was mentioned to be mainly apparent for the cognitive task demands (eg, game speed and task complexity), whereas physical exercise intensity is often (too) low and could be increased (f=4):

For MCI-patients, some games are predestined to be used with them, such as “Simple,” “Flexi,” “Birds” and perhaps also “Habitats.” These games don’t put so much time pressure and the feeling of having missed something on patients.

E-08: project manager therapy

[...]the increase in the challenge profile from the easiest games (“Simple” and “Birds”) to the next more difficult game is too steep for MCI-patients. For example, the game “Targets” is too fast for many patients. The game “Habitats” contains too many stimuli at once, so that the patients no longer know what they have to pay attention to.

E-07: physical and occupational therapist

[...]I have the impression that the internal progression, which is responsible for adapting the game demand, sets the lower limit too high and adapts too quickly, so that the cognitive overload becomes visible very quickly, especially in MCI-patients.

E-08: project manager therapy

One problem with the “Senso,” in general, is that the physical intensity might well be higher.

E-05: neuropsychologist

Overwhelming task demands may cause frustration or refusal of games (f=6), although the feedback mechanisms to indicate errors work subtle (f=4). In contrast, games that are perceived as being too easy lead to boredom (f=2):

For example, the games “Targets,” “Ski” or “Hexagon” are very confronting, and patients recognized quite quick: “Okay, I can’t do it,” and that frustrates patients. [...] Usually, these patients stop in the middle and say something like: “Ah, I don’t need that kind of shit.” Most of the time, they stop the training session immediately and don’t want to continue anymore.

E-08: project manager therapy

My observation was that the negative feedback currently used does not demotivate the patients at all. It is also clear to the patients that they need to know when they are making mistakes and whether they are completing the tasks correctly.

E-04: exergaming researcher

Some of the negative feedback is so subtle that it is not even noticed.

E-05: neuropsychologist

#### T3: Motivators for Training

The experts described numerous motivators for training older adults with mNCD. The most frequently described motivators can be classified as intrinsically regulated motivators (f=44), which are directly related to exergames. Excitement, enjoyment, or fun is perceived as a central motivator for performing exergames (f=4). This was reported to be maintained by the captivating character of exergames (f=1) and multimodal animation (f=1), which is supported by specific game components (eg, game tasks or designs close to everyday life [f=6] or with personal relations or memories [f=1] including music or sound effects [f=8], animals or plants [f=4], landscapes [n=1], or colors [f=1]). In addition, patients were described as intrinsically motivated by gamification (f=6), the feeling of being optimally challenged (n=3), or simply by the variation of training (f=6):

For patients, the focus is primarily on having fun with the games. For example, they [...]liked watching birds and listening to birdsong and felt very motivated by the personal connection. Through these personal memories [...] a whole other level of motivation emerged.

E-08: project manager therapy

I think that those people who enjoy playing games are generally captured by the playful and competitive nature of the games. Furthermore, training with exergames is something completely different compared to classical therapy. Patients appreciate this change from the “dry” standard therapy.

E-06: physical and occupational therapist

However, when task demands become too high (f=6) or too low (f=2), patients have been observed to promptly lose their willingness to perform the exergames, as already reported. External motivators such as social support (eg, by therapists or caregivers) or group dynamics have also been reported to improve motivation to train (f=12). Feeling concerned about cognitive deterioration or being confronted by performance classifications can either motivate or induce negative feelings (f=7). Finally, some patients were also reported to be motivated by the effectiveness of exergames (f=2) or performance improvements (f=2):

I consider this social support to be very central. [...] If a relative joins in for motivation or support it can be very valuable.

E-04: exergaming researcher

I think there are always patients who don’t want to know how well they are performing. Forcing performance feedback on such people can of course be motivating, but it could also be negative and confirm their limitations.

E-07: physical and occupational therapist

From the patients’ viewpoint, all patients reported that they could primarily be motivated to train regularly by the effectiveness of the training, helping them achieve their individual success (f=13, n=8, 100%). Alternately, patients reported being motivated by their relatives or partners (f=2, n=1, 13%) and enjoyment of exercising (f=1, n=1, 13%). Having to travel to a training facility was reported to have a negative effect on training motivation and adherence (f=4, n=1, 13%):

It would be nice if I could go for a walk in the forest again without falling down. I used to do this four times a week for 75 minutes. It motivates me to train so that I can do this again in the future.

P-02

It would motivate me if I could improve my abilities (balance) again. [...] I would like to stay independent and modern, not to be called an old lady.

P-03

The success. I no longer need to be motivated. If I set my mind to it, I do it!

P-08

#### T4: Training Goals and Outcomes

Regarding the training goals, cognitive functioning (f=19) should be targeted in the training intervention in the experts’ viewpoint while also addressing ADLs and mobility (f=3), addressing physical capabilities (f=3), and accounting for psychosocial factors (f=2), such as feelings of insecurity. However, the weighting of the training focus differs significantly between experts in different fields:

[...] higher order processes (i.e. divided attention or the ability to plan) are affected in most patients. Therefore, it is important to focus on these higher order cognitive functions.

E-05: neuropsychologist

I think that the coupling of brain functions with physical functions is central. At the same time [...]it is important to focus on what is impaired. If the frontal lobe is impaired, it is certainly important to train executive functions, attention and inhibition.

E-10: founder of an exergaming company

Primarily physical activation, especially that people get moving and walk. But also, to train the intuitive way of taking steps. [...] The cognitive aspects of the training have always played a subordinate role for me, but they were usually not decisive for the success of the therapy, as this was often trained differently, and I am not an expert in this.

E-06: physical and occupational therapist

When asking experts about the training goals of patients, ADLs and mobility (f=5) were the most frequently stated in addition to cognition (f=3) and physical functioning (f=2). In addition, psychosocial factors (f=2) have been reported to include socializing or having fun:

I had patients who wanted to continue training because the training made them more confident in their gait. They felt better balance after the training.

E-06: physical and occupational therapist

The patients also see the cognitive aspects of the training, of course. [...] We often explain to the patients that falls prevention has a cognitive and physical aspect and that these aspects interact. Therefore, the patients mainly go to the training with the aim of improving their gait.

E-07: physical and occupational therapist

Some people really know what’s going on and they know that they have a disease and that they can prevent or slow down the progression by doing physical activity and exergames. But then others don’t really know that they have cognitive deterioration and they’re just playing a game and having fun without specific training goals.

E-09: exergaming researcher

This is consistent with patients’ viewpoint who most frequently reported improving gait (f=6, n=5, 50%), memory (f=3, n=3, 38%), and balance (f=2, n=2, 25%) as their primary goal to increase their quality of life. In addition, patients reported being more active (f=1, n=1, 13%), increased functional abilities (ie, cooking; f=1, n=1, 13%), increased strength (f=1, n=1, 13%), or remaining independent in ADLs (f=1, n=1, 13%) as training goals:

It is mainly the memory. It is memory because it affects a lot of other things.

P-01

It would be wonderful, if I could go for a walk in the forest again without falling down.

P-02

I really want to remain independent. I definitely don’t want to become dependent on others.

P-05

That I can keep things better in my head. That has diminished. That would be nice!

P-06

I want to have more strength again to increase stability and be able to walk longer.

P-08

#### T5: Exergame and Training Components

##### Location

Regarding training location, the experts reported that the patients would either prefer individual training at home (f=3) or in a mixed setting, including training at home combined with training at a clinic (f=4). None of the experts stated that patients would prefer exercising at a clinic or training facility in general, as this is often associated with excessive time expenditure. Training at home was reported to be beneficial, because it represents a known environment that makes patients feel more secure. However, the experts also stated that patients may not be capable of performing exercises or exergames independently and therefore need guidance throughout each training session (f=4) or at least partially (f=9); for example, when starting up the system or in case of technical problems:

The advantage of training at home is that “it’s a known environment and they feel safer at home and also don’t have to travel.” However, “I would suggest that the help of a guiding therapist with experience will be necessary.”

E-09: exergaming researcher

In a previous investigation [...], patients’ feedback was that 70% could imagine doing the training from home. [...] For MCI-Patients specifically, relatives may be involved. But in general, the need for home-based exergame training is there, I would say.

E-08: project manager therapy

This is also reflected in the outcomes of the question of whether patients would be capable of performing home-based exergame training; the experts mainly reported that patients are certainly capable (f=4) or should be capable of considering some concerns (f=9) to perform such a training program independently at home. The concerns that need to be considered include the improvement of game instructions (f=2), accessibility of a handrail or similar for safety support (f=2), and avoidance of technical problems (f=2) or the integration of a guided familiarization period (f=1) or support of a care professional or partner (f=2):

I think if the system would really work properly then you could use it at home. However, if you just have some minor technical problems is already like a no-go to use it at home at all.

E-01: exergaming researcher

It would certainly be good if the patients could complete an accompanied training for a certain period of time in order to facilitate the transfer to training at home.

E-04: exergaming researcher

[...] some kind of adjustment of the instructions is needed [...], especially for this patient group and for independent training in the home-based setting. [...] The instructions have to be adapted in such a way that understanding can be achieved without someone having to stand next to the patients all the time.

E-10: founder of an exergaming company

Of those patients who responded to the question and had a clear preference regarding the training location, most (6/7, 86%) patients would clearly prefer to train individually at home, because it is less time consuming and more flexible. One patient did not have a clear preference; she simply wanted to perform the exercises where it was easiest for her and preferred group exercises:

For me, it is important that the training can be done independently at home. If I have to go to the doctor somewhere every time, it’s simply too much work.

P-01

Preferably at home, if I can. Then I can also choose the time when I want to exercise. I have lived my whole life with a packed schedule. Now I want to be a little freer and more flexible.

P-03

##### Safety

The experts reported an increased risk for falls, as patients with mNCD (1) are easily distractable and (2) have difficulties in self-assessment and impaired planning abilities. Therefore, it was recommended to use the handrail in the beginning to minimize the risk of falls (f=3), which was also requested by 1 patient. In the case of a home-based exergaming system—which may not have a handrail—thorough and clear safety instructions are recommended (f=1):

Especially in the beginning, until the patients have understood what it is all about, it is very important to instruct using the handrail.

E-04: exergaming researcher

I definitely need a railing to prevent falls during training. I often fall down if I don’t have anything to hold on to.

P-02

##### Instruction, Familiarization, and Guidance

As illustrated earlier, certain adaptations are required to enable a more independent use of the exergaming device. First, patients should be familiarized with the exergaming device and the corresponding games considering the following key elements: (1) start at an easy level (f=7), for example, by using the game “Simple” (f=4), (2) ensure that patients voluntarily try out the device (f=3), (3) ensure that you are not too confronting (f=2), (4) give patients enough time to familiarize with the new task (f=1), and (5) start with a reaction game, then progress to games for specific domains of neurocognitive function (f=1):

It is very important to start very slowly and at a low difficulty level until the patients can better assess their abilities on the “Senso”. […] Since the game “Simple” waits for a reaction from the individual, it is very suitable to start with.

E-04: exergaming researcher

We always start with a reaction game so that the patients can learn the coupling of the cognitive-motor functions and learn to interact with the environment. Later on, we focus on specific cognitive functions.

E-10: founder of an exergaming company

Regarding the instructions, some adjustments are needed to improve comprehensibility. Currently, there is instructional text before starting each game. However, patients with mNCD have been reported to have limited comprehension of instructions. Therefore, adaptations are needed in the instructions of exergames in general and for a home-based exergaming system in particular. The experts mainly suggested to use step-by-step (f=3) instructions based on a combination of visual (ie, written instruction or video demonstration) and verbal instructions (f=4) guided by an experienced therapist (f=1). In case of more severely impaired patients or for home-based exergaming systems, it was suggested that practical demonstrations (f=2), video instructions (f=6) or even interactive “trial run” instructions (f=5) could improve comprehensibility of the games:

The transfer from the written instructions to the understanding of what is to be done in the game is sometimes difficult.

E-06: physical and occupational therapist

Personally, I would replace the written instructions with a short (few seconds) video sequence showing the most important functions of the games.

E-08: project manager therapy

I would recommend combining visual and verbal instructions. For example, through a visual presentation with additional step-by-step verbal instructions. Verbally we can “pick up” the patients very well and get a feeling whether the patients have understood the instructions.

E-03: neuropsychologist

[...], some kind of adjustment of the instructions is needed. [...] It is definitely important to pursue and use these adaptations, especially for this patient group and for independent training in the home-based setting”, because “in the case of more severe impairments, it is often necessary to demonstrate the games step by step by yourself. [...] In other gaming systems there is a short test phase with explanations and trial runs [...]. However, this would have to be offered as an option, since most patients will no longer need it after a few sessions.

E-10: founder of an exergaming company

Finally, when guiding patients through their training sessions, social support and guidance by a care professional or partner might be beneficial (f=3). However, it was also mentioned that this might be critical because of personal conflicts or patients’ psychological constraints (f=2):

Family members could play an important role in reminding and motivating patients to complete their training.

E-06: physical and occupational therapist

I don’t think it’s always a good idea to include family members as guidance, because the pressure to perform gets higher for the patients, since they try to hide their impairments from others. A health care professional like a nurse for example or physical therapists would be better than a husband or wife, I think. They already have a lot of fights in the households, because things are not working out as they should.

E-09: exergaming researcher

From a patient’s perspective, all patients reported that they can imagine training alone, provided they had received thorough instructions and understood their tasks. One patient additionally requested regular support from a care professional or partner:

Yes, I think so. Once I learn that, I’m sure I can do it independently.

P-03

If I am supported by you or by my partner, then I can certainly train partly independently.

P-07

##### Exergame System and Content

Previous experiences of older adults with mNCD using the exergaming system “Senso” are illustrated earlier. Building on this, several game-specific adaptations were suggested by the experts (f=9):

More time should be provided between the balls so that the flood of information is reduced (it is often overwhelming when several balls are visible on the screen very quickly).

E-07 (physical and occupational therapist): for the game “Targets”

In the initial phase, until patients’ have understood all the game tasks [...], the speed must definitely be reduced.

E-06 (physical and occupational therapist): for the game “Habitats”

There are already enough opportunities to increase the task difficulty. [...] However, it is very important to note that the game difficulty is adjusted downwards so that it is easier to start the training.

E-08: project manager therapy

In addition to these game-specific adaptations, multiple novel game designs and elements have been suggested and discussed by focus groups to address patients’ needs optimally. In general, it has been recognized that there is a need for new games specifically targeting the neurocognitive functions of learning and memory (f=4) and executive functions (ie, working memory and cognitive inhibition; f=2). Specific game design suggestions were discussed for such a memory or working memory game. Additional suggestions for new game designs and elements include the use of music, addition of visual reminders to guide patients within the games, or adaptations in performance feedback:

With the “Senso”, a certain spectrum of neurocognitive function domains is covered. However, games for working memory, inhibition or memory are completely missing. In the case of memory, there is currently only one game available specifically targeting the training of short-term memory span.

E-05: neuropsychologist

I think music would be very motivating for people with MCI or dementia also if is music from their youth or music they like. It’s also been described in the literature that music has so many good effects on people when they have heard a song that they liked before and they are singing that song.

E-09: exergaming researcher

In addition, it would be good to include reminders, for example at the edge of the screen, which patients can use for orientation. [...] Additionally, [...] it would certainly be helpful here if the program not only displayed the performance curve, but also provided a reason or explanation.

E-03: neuropsychologist

As general requirements when designing new games, the experts recommended using simple graphics and ensuring good contrast (f=14), a comfortable relation, and good usability of the exergames (f=4) using easily comprehensible and clearly designed tasks (f=2) with a certain closeness to everyday life (f=7). Multimodal animations, including multisensory feedback (f=7), should additionally be integrated by focusing on positive reinforcement mechanisms (f=2) to motivate the patients during exergaming. In addition, it is important that the main task is in the center of the screen (f=1) and that only elements that are related to the game task are included (f=5). Moreover, too confronting performance feedback (f=1) and unexpected appearance or technical problems (f=2) should be avoided:

It is very important to create a good contrast. [...] It’s generally important for the older population to keep the graphic representation as simple as possible, because for older people, the game is not characterized by great graphics, but by the game task as such. The main importance is that the right level of challenge is offered.

E-08: project manager therapy

It is much better to present a simple graphic and focus on the aspects that need to be trained. [...] unnecessary graphic gimmicks should be avoided!

E-04: exergaming researcher

It is important to have a main action that is in the center of the screen and to ensure that the player will have primary task in the center. If you put any secondary tasks into the games, it can be confusing for the patients.

E-02: exergaming researcher

Spontaneously, I would say that games close to everyday life are more popular. [...] These games were much better received than abstractly structured games (“visual exploration tasks”).

E-03: neuropsychologist

My experience so far is that games that are designed to be more relevant to everyday life (and simpler) work better. Therefore, new game designs should be based on what patients know from their everyday lives.

E-06: physical and occupational therapist

##### Training Components

The recommended exercise frequency ranged from 2 (f=3) to 5 or more (f=4) training sessions per week, largely dependent on training location and motivation. The recommended session durations ranged from a maximum of 15 to 20 minutes (f=3) up to 30 minutes (f=2), with the aim of reaching a moderate exercise volume of approximately 150 minutes per week (f=1). Shorter sessions and a higher training frequency have been reported to be preferable to reach this training volume, mainly owing to attentional exhaustion:

The more the better! I would prefer shorter training sessions, especially because of attentional exhaustion. Here I would recommend a maximum of 30 minutes and at least 5 sessions a week. This is much better than training for 2 hours at a stretch!

E-03: neuropsychologist

I would recommend a training frequency of 2 – 3x/week. [...] The training duration is difficult to estimate. Some patients are already exhausted after 2 minutes, others can easily train for 20 minutes.

E-10: founder of an exergaming company

I think that a training frequency of 3x/week is already (too) much. 2x/week should be possible to arrange. 1x/week definitely works. This may be because three appointments, in combination with other activities, may already be too much for patients. If the training could be done at home, the training frequency could certainly be increased up to 4 - 5x/week. In this case, motivation could still be difficult.

E-07: physical and occupational therapist

I would aim for a training volume of 150 min/week. As far as I know, this is considered moderate for older patients. I would consider 100 min/week as the lower limit. A minimum of 3 x per week for 30 min would also be okay at best.

E-08: project manager therapy

Exercises requiring a coupling of physical and cognitive functions were described as preferable and should be prescribed domain-specific depending on the patient’s abilities:

I think that the coupling of brain functions with physical functions is central. Whether this is ultimately an attention game, or a training of the executive functions is something I don’t consider central at the beginning. Of course, it also plays a role here which cognitive functions are impaired. [...] If the frontal lobe is impaired, it is certainly important to train executive functions, attention and inhibition.

E-10: founder of an exergaming company

To maintain the training program in the long term (preferably >12 weeks), motivation is a key factor that can be facilitated by the playful character of the exergames and a variation in the choice of games. Nonetheless, patients seem to prefer a certain routine:

Of course, the training should be maintained over a certain amount of time at a stretch. So not just two weeks, but ideally longer (more than 12 weeks). Of course, motivation is also a very central point. If the training is varied and has a playful character, this should be feasible.

E-03: neuropsychologist

Patients are generally routine-oriented, which can also be observed in general. Therefore, it is also important to introduce a new game every now and then. The patients primarily prefer the familiar games and should therefore be challenged to a certain variety.

E-10: founder of an exergaming company

The physical exercise intensity should be maintained at a light to moderate level, while the focus should be on game complexity that should be challenging but feasible. Game complexity can be varied on multiple levels, for example, (1) stability support (use of handrail with both hands, 1 hand, or no support), (2) stepping direction, (3) game choice and tasks included, (4) game duration, or (5) game speed:

Adding new games. I always start with the game “Simple” and sometimes in the first session I also introduced “Birds” when I think it would be possible. If not, then I will do it the next session. If somebody is really performing well and understanding all the instructions, then I also progress to the game “Targets” and even “Birds”.

E-09: exergaming researcher

I also often started with just stepping movements forward [...] and included the step direction to the right at a later timepoint.

E-10: founder of an exergaming company

We have a routine that we usually do the training sessions over 3 weeks and do the first 3 sessions with holding, just to get a feel for the games. After that, we gradually go back to holding on with one arm and without holding on.

E-08: project manager therapy

From the patients’ viewpoint, a high training frequency (mean preferred training frequency 5.21 times per week; n=7), ranging from 2 times per week (n=1, 13%) to daily sessions (n=4, 50%)) with short session durations (mean preferred session duration 23.4, SD 10.3 minutes; n=8), ranging from 10 minutes (n=1, 13%) up to 30 minutes (n=3, 38%) was preferred. Five of 6 (83%) patients who responded to the questions about how long they would prefer to do the training stated that they would prefer to continue the training as long as they profit from it and are able to do it. All patients preferred a training that is individually adapted to apply moderate (4/5, 80% of patients) to high physical (1/5, 20% of patients) intensity and moderate (3/5, 60% of patients) to high (2/5, 40% of patients) cognitive challenges:

If the device was at home, I would do the training every day.

P-01

I don’t want to make a guarantee now, but I could do a short training session every day for like 20 minutes or so. But I can’t promise that I’ll do 40 minutes every day, because I also want to do other things. Especially when the weather is nice, I like to go outside. And then I also must do the housework, which also takes time.

P-03

I think about 30 minutes is good. If it goes on too long or is too strict, then I get tired of it. I don’t like that. That would be counterproductive.

P-05

If I have the device, I could do this training forever. As long as I still have the strength to do it.

P-02

I would need a bit of a start-up period first. If it’s not effective, I’ll stop again. Additionally, I don’t know how my health will be in the future. But as long as I’m reasonably fit, I’ll definitely want to do it.

P-07

##### Individualization

Individualization of the exergame intervention concept should mainly account for two aspects: (1) task type (ie, choice of exergames to individually focus on neurocognitive functioning; f=4) and (2) task demands (ie, adapting the game demands according to the individual capabilities to maintain a challenging but feasible cognitive load; f=5). In addition, it was recommended to change between games with different task demands to enable the maintenance of attention over the entire training duration (f=2) and to supervise training exertion (f=3):

It is important to have a system that will adapt the games according to the participant’s performance.

E-02: exergaming researcher

The physical intensity is often not a problem, and it should primarily be the complexity of the training that is individually adapted so that it is doable and still has a certain physical demand.

E-10: founder of an exergaming company

[...] We also have to check whether somebody is very fatigued [...]. Sometimes you have to let someone take a rest because they will not always feel when they have to take a rest.

E-09: exergaming researcher

One should “[...]alternate between games that focus primarily on performance and less on cognitive aspects with more cognitively demanding games.”

E-08: project manager therapy

## Discussion

### Principal Findings

The objective of this study was to determine the capabilities, treatment preferences, and motivators for training older adults with mNCD, as well as their perspectives on training goals, settings, and requirements for exergames and training components. This will—together and in line with a synthesis of the optimal evidence-based informed decisions—serve as basis for user modeling, determination of therapeutic needs, and definition of a set of requirements for the game design and development process of a novel exergame-based training concept. To the best of our knowledge, this is the first study to systematically and thoroughly investigate user requirements and preferences for an exergame-based training concept before it is designed and developed specifically for older adults with mNCD based on these findings.

The results of our qualitative study, which included focus groups with 10 experts or health care professionals and individual semistructured, in-depth interviews with 8 older adults with mNCD, yielded the following key findings: (1—capabilities) from a patients’ viewpoint, the psychosocial consequences of their self-perceived cognitive deteriorations might be more burdensome than the cognitive changes themselves; (2—treatment preferences) more integrative forms of training (such as exergaming) including gamified tasks close to everyday life, multimodal animation, and acoustic feedback are preferred by patients. None of the interviewed patients reported any previous experience with exergaming, but all patients described the handling of different technologies as feasible despite some challenges and were willing to try out exergaming; (3—motivators for training) from the expert’s viewpoint, the most frequently described motivators to train can be classified as intrinsically regulated motivators such as excitement, enjoyment, or fun in exercising that is maintained by the captivating character of exergames supported by specific game components (eg, game tasks or designs close to everyday life or with personal relations or memories including music or sound effects, animals or plants, landscapes, or colors); the feeling of being optimally challenged; and the variation of training. All patients reported that they could primarily be motivated by the effectiveness of the training, helping them to achieve success on an individual basis; (4—training goals and outcomes) the most important training goals of older adults with mNCD include improvements in ADLs and mobility (gait and balance) and memory, because these outcomes were described as central to improving their quality of life; (5—exergame and training components) the use of home-based exergames as a form of simultaneous-incorporated motor-cognitive training should be prescribed with a domain-specific training focus depending on a patient’s cognitive abilities, a high training frequency (4-5 training sessions per week), short session durations (20-25 minutes), and individual adaption and progression of task type and demands to reach a light to moderate level of physical intensity and a challenging but feasible game complexity. To maintain the training program in the long term (preferably >12 weeks), motivation is a key factor and should be facilitated by the playful character of the exergames, variation in the choice of games, and ensuring that the patients are optimally challenged. To make home-based training interventions feasible, multiple factors that need to be considered were identified. Patient-friendly game instructions are needed, while the accessibility of a handrail or similar for safety support, avoidance of technical problems, and the integration of a guided familiarization period or support from a care person need to be ensured to make home-based exergame training feasible. As general requirements for exergame design, simple graphics with good contrast and easily comprehensible and clearly designed tasks with a certain closeness to everyday life should be used. Multimodal animations, including multisensory feedback that focuses on positive reinforcement mechanisms, should be integrated to motivate patients during exergaming. In addition, it is important that the main task be in the center of the screen and that only elements that are related to the game task are included. Moreover, confronting performance feedback and unexpected appearances or technical problems should be avoided.

### Capabilities of Older Adults With mNCD

A variety of cognitive changes mainly affecting the neurocognitive domains of learning and memory, complex attention, and executive function were discussed by the focus groups and mentioned by the patients, whereas no serious restrictions on physical capabilities, mobility, and ADLs were reported. This is in line with DSM-5 [9]. According to the definition of mNCD, modest (ie, for mNCD, performance typically lies in the 1-2 SD range) deterioration in cognitive functioning can be observed, whereas the capacity for independence in everyday activities is preserved [9]. However, from the patient’s perspective, the most prominent consequences of their disorder were described as affecting psychological factors, mainly by causing psychological distress, feelings of insecurity, and depression. It is well known that depression and anxiety are common in older adults with mNCD [72,73]. In addition, patients with depression have higher rates of conversion to dementia, indicating that depression is an important risk factor for cognitive decline and progression to dementia. This emphasizes the importance of assessing depressive symptoms in older adults with mNCD [72].

### Treatment Experience and Preferences

Most of the interviewed patients had already gained experience with different treatment or training approaches to counteract cognitive decline and preserve physical capabilities, mobility, and ADLs. Although MTT, physiotherapy, and CCT were perceived as useful, the patients reported being insecure about the effectiveness of these approaches or that they would have to (be able to) do it more consistently to profit from it, which was described to be limited by the availability of therapists. More integrative forms of training, including gamified tasks close to everyday life, multimodal animation, and acoustic feedback, were reported to be preferred by patients.

This is in line with the literature, showing that *“*research involving older adults has found that CCT programs are associated with high satisfaction levels, and that they are also a feasible option for individuals with MCI, with equal or better adherence rates when compared with traditional cognitive training*”* [74-76]. This is also evident in the use of exergames. Exergame-based training interventions are widely accepted in individuals with mNCD, and exergames increase or enhance participants’ motivation to engage in rehabilitation activities [40]. This is also reflected by the adherence rates to different types of exercises in patients with mild to major NCD. Recent systematic reviews and meta-analyses synthesized mean adherence rates of 70% [77] for physical exercise interventions, whereas the mean adherence rate was higher for exergame-based interventions at 84% [78]. To the best of our knowledge, there is no systematic review that has synthesized adherence rates to CCT. However, Turunen et al [79] investigated adherence to a long-lasting multidomain CCT among a sample of 631 older adults at risk of dementia. It was shown that only 20% of participants completed at least half of their CCT sessions, and only 12% of participants completed all (maximal number of training sessions=144) of their training sessions. In addition, 37% of the participants did not train at all, whereas *“*previous use of computers, better memory, being married/cohabiting, and positive study expectations were independently associated with the greater probability of starting the CCT. Previous computer use was the main determinant of the number of CCTs completed after the training was initiated*”* [79]. Therefore, when comparing these findings, it appears that exergame-based interventions have the highest adherence rates among different training programs. This is consistent with findings in HOAs, where adherence to technology-based training programs was higher than that to traditional training programs, independent of study site or level of supervision [80]. This finding may be largely explained by the high level of enjoyment in using technology-based physical exercise programs [80]. Technology-based training systems offer several advantages over traditional training programs that may contribute to a more enjoyable exercise experience. For example, exergames can provide real-time feedback and positive reinforcement while exercising and can monitor performance over time [80]. In addition, exergames enable individual real-time adaptivity of physical and cognitive task demands according to the participants’ performance or physiological response (eg, heart rate and brain activity), which is considered a key advantage of serious video games (such as exergames) [81-83]. In fact, the findings of our study suggest that applying an optimal challenge is central to promote the use of exergames in patients with mNCD in the long term.

When considering the experts’ previous experience in the use of exergames (ie, “Senso”) with patients with mNCD, the interaction with the device, its overall usability, and the design of the exergames were described as good. Especially the simple and clear game design structures were reported to be highly appreciated by patients and to promote good task comprehensibility. Various minor usability issues were reported, including difficulties in the interaction with the exergame training system “Senso” (eg, unintendingly walk off the middle plate without noticing the feedback on the screen), but mainly, usability issues that related to capabilities of older adults with mNCD (eg, limited comprehensibility of the game instructions) were reported. These usability issues need to be considered and addressed when developing a training concept specifically for older adults with mNCD. Nevertheless, it is important to emphasize that these are only minor usability issues, and only minor refinements are required to optimize the exergame experience. This is also illustrated by recent studies showing that exergame-based training programs using the “Senso” are feasible; usable; and widely accepted in different populations including community-dwelling older adults [84], geriatric inpatients [51], and patients with major NCD [52], chronic stroke [85], or multiple sclerosis [86]. Therefore, when designing and developing an exergame-based training concept specifically for older adults with mNCD, these refinements should primarily target the adaptability and individualization of task demands and the optimization of the instruction of the exergames.

### Motivators for Training

The motivating factors most frequently described by experts were classified as intrinsic motivators. These were described as being maintained by the captivating character of exergames and promoted by specific game components such as game tasks or designs close to everyday life or with a personal relation or memory, including music or sound effects, animals or plants, landscapes, or colors. In addition, patients were described to be intrinsically motivated by gamification and the feeling of being optimally challenged. From a patient’s perspective, the effectiveness of the training, which helped them achieve their individual success, was clearly the most prominent motivator.

This is consistent with reports in the literature. More autonomous forms of motivation can be promoted by various factors, although these factors may vary depending on the population. For example, a small case-control study compared the motivational factors for using a balance exergame platform between healthy younger and older adults. It was shown that *“*older adults were more intrinsically motivated by the joy of playing and extrinsically motivated by the perceived health effects (physical and cognitive), with less regard for the in-game rewards*”* [87]. To provide effective interventions to promote physical activity [88] in patient with NCDs, a new theoretical model has recently been introduced. This theoretical model is based on the review of existing theories that explain behavior change in relation to physical activity in HOA, which were then adapted and integrated to a new theoretical model called the “PHYT in dementia” [88]. In this framework, several additional key elements for promoting behavioral changes in physical activity have been proposed. These consist of self-efficacy, including embarrassment (eg, supervision of activity had a negative impact on engagement in the intervention), personal concerns (eg, fear of falling), and routine (eg, flexible integration of physical activity intervention into daily life regarding place and time of performance), as well as appropriate challenges [88]. A detailed awareness of participant motivators is required, especially for the preference that the routine can be performed at home and at different times during the day [88], because self-determined motivation may be a central aspect of adherence to home-based training programs [89].

### Training Goals and Outcomes

The interviewed experts recommended to mainly target cognitive functioning when developing a training concept for older adults with mNCD, while ADLs and mobility, physical capabilities, and psychosocial factors should also be accounted for. This is consistent with the patients’ viewpoint that most frequently reported improving gait and memory as their primary training goals to increase their quality of life.

Similar results have been documented in the literature. According to a survey of patients who completed a multicomponent behavioral intervention for patients with MCI and their caregivers, quality of life was the most important outcome priority for patients with MCI, followed by self-efficacy, depression, basic ADLs, memory-based ADL, anxiety, and memory performance [90].

### Exergame and Training Components

The use of exergames as a form of simultaneous-incorporated motor-cognitive training is recommended, which should be prescribed domain-specifically, depending on a patient’s cognitive abilities. Previous studies applying exergame-based motor-cognitive training in older adults with mNCD or MCI have used commercially available exergame systems [76,91-96] or exergames that were specifically developed for patients with mNCD or MCI [97-100], which comprised sensor-based stepping platforms [94], video camera–based or wireless remote device systems [76,91,95,97,99], or exergames that were controlled using a cycle ergometer or similar [92,93,96,98,100]. The training programs can be classified as simultaneous- additional [92,93,96,98] or simultaneous-incorporated [76,91,94,95,97,99,100] motor-cognitive training that was applied targeting 1 [93,100] or multiple [76,91,92,94-99] neurocognitive domains, including complex attention [76,91,92,94-99], executive functions [76,91,92,94-100], learning and memory [91,93-95,97-99], or visuospatial skills [76,97,99]. Only one of these studies applied training that individually prescribed content on the basis of a patient’s cognitive abilities [96]. However, it has not been performed or reported in a reproducible manner.

Therefore, so far and to the best of our knowledge, 11 studies have been published that investigated exergame-based motor-cognitive training in older adults with mNCD or MCI. Most of these studies designed or used exergames that could be classified as simultaneous-incorporated motor-cognitive training. Incorporating cognitive tasks into motor tasks may be more beneficial for consolidating neuroplasticity [42], because (1) it leads to greater (motor) cognitive improvements, (2) it is closer to daily life situations, (3) no prioritization effects occur, which can be observed in motor-cognitive training with additional cognitive tasks, and (4) multiple sensory systems are stimulated at the same time, which may provide an optimal basis for cognitive processes such as learning [42]. Meta-analytic evidence suggests that simultaneous motor-cognitive training is the most effective type of training for improving cognition in HOA [43,44] and in older adults with mNCD [44-46]. Nevertheless, it remains to be evaluated whether the incorporation of cognitive tasks into exercise or training interventions indeed results in more distinct effects on cognitive performance compared with simultaneous motor-cognitive training with a non–task-relevant secondary cognitive task [42]. Finally, there seems to be room for improvement regarding the domain-specific prescription of the training content, considering a patient’s cognitive abilities and the adaptation and development of exergames specifically for patients with mNCD. This may be especially relevant when considering the large heterogeneity in the clinical symptoms of older adults with mNCD. Remarkably, most previous studies applying exergame-based motor-cognitive training in older adults with mNCD or MCI have used commercially available exergame systems [76,91-96], in which the training content does not specifically target patients with mNCD. This is consistent with the findings of HOA. In a systematic review, Valenzuela et al [80] emphasized that in HOA, most studies used commercially available exergame systems. It was argued that these systems may be difficult to use for those with little or no experience with technology, because these systems often lack clear instructions, present too much graphical information, and have not been designed and developed to provide optimal training components for the target population and aims of the studies in which they were used [80]. In fact, all previous studies applying exergame-based motor-cognitive training in older adults with mNCD or MCI have used exergames with complex 2D or 3D virtual environments [76,91-100]. This may not be optimal because the limitation that such systems may be difficult to use for those with little or no experience with technology could be even more pronounced in patients with mNCD, as these patients are easily distracted and quickly overwhelmed by the task demands. Indeed, according to the recommendations of the interviewed experts, it is beneficial to focus on the aspects that need to be worked on by implementing easily comprehensible and clearly designed exergame tasks and to only present elements that are directly related to the game tasks while avoiding unnecessary graphical information or distractors.

According to the recommendations of the interviewed experts, the training program should be maintained over the long term (preferably ≥12 weeks). A training frequency of 2 to 5 or more training sessions per week was recommended, largely depending on the training location and motivation. In addition, it is recommended to reach a moderate training volume of approximately 150 minutes per week. To reach this training volume, shorter training sessions and a higher training frequency should be applied, because longer training sessions might lead to attentional exhaustion in this group of patients. Therefore, the experts recommended session durations between 15 and 20 minutes up to a maximum of 30 minutes. Previous studies applying exergame-based motor-cognitive training in older adults with mNCD or MCI have prescribed training programs over durations of 5 weeks [97], 6 weeks [91,94,98,100], 12 weeks [96,99], 3 months [93,95], 24 weeks [76], or 6 months [92]. The prescribed training frequency was 1 time per week [76], 2 times per week [94,96,99], 2 to 3 times per week [95], 3 times per week [97,98], 3 to 5 times per week [92,93,100], or 5 times per week [91] with session durations of 15 minutes [99], 18 to 30 minutes [94], 20 to 80 minutes [97], 25 to 30 minutes [91], 20 to 40 minutes [93], 30 to 45 minutes [100], 40 to 45 minutes [98], 45 minutes [92], 60 minutes [96], 90 minutes [76], or not reported [95], resulting in a weekly training volume of 30 minutes [99], 36 to 60 minutes [94], 60 to 200 minutes [93], 90 minutes [76], 90 to 225 minutes [100], 100 to 145 minutes [97], 120 minutes [96], 120 to 135 minutes [98], 125 to 150 minutes [91], 135 to 225 minutes [92], or not reported [95]. Therefore, most of these studies prescribed a training volume that was in line with the recommendations of the experts in this study. However, the session durations often exceeded the experts’ recommendations, whereas the training frequency was lower than recommended. To avoid attentional exhaustion of the patients during training, future training concepts might consider prescribing shorter session durations while increasing the training frequency to achieve a similar training volume per week. This might actually improve the effectiveness of the intervention because higher training frequencies have already been shown to promote the effectiveness of physical (ie, ≥4 times per week) [101] and cognitive training (ie, >3 times per week) [102], while shorter session durations (ie, ≤30 minutes) [101] of physical exercise have been shown to exert more pronounced training effects. These findings might also apply to simultaneous motor-cognitive training. A meta-analysis revealed that training frequency is a significant moderator of the effects of physical and motor-cognitive training interventions on cognitive functioning, favoring higher training frequencies (≥5 times per week) in a mixed population of HOA and patients with mNCD [103]. Finally, a high training frequency (approximately 5 times per week) with short session durations (approximately 20 minutes) would also match the preferences of the interviewed patients in this study.

The experts reported that the training should preferably be individually carried out at the patients’ homes, not only because it represents a known environment that makes patients feel more secure and represents a less-confronting environment for them (because they do not have to hide their impairments from others when training alone), but also to allow higher training frequencies. Nonetheless, to ensure that training in patients’ homes is feasible, multiple factors need to be considered. For example, improvements in game instructions are required, a handrail or similar needs to be made available to allow safety support during training, and technical problems must be avoided. In addition, a guided familiarization period and part-time supervision or support from a care professional or partner should be integrated to make the transfer to home-based exergaming easier. Previous studies applying exergame-based motor-cognitive training in older adults with mNCD or MCI have administered individual [91] or group-based [76,96,99] training sessions, and the training setting (ie, individual vs group sessions) has not been clearly reported [92-95,97,98,100]. The training sessions were conducted at the hospital [91], in a nursing home [94], at day-care centers or memory clinics [99], at a centrally located church [76], at patients’ homes [93,100], or the training location was not clearly reported [92,95-98]. The training sessions were supervised by a therapist [91,94,96], or supervision was not reported [76,92,93,95,97-100]. Consistent with summarized previous studies applying exergame-based motor-cognitive training in older adults with mNCD or MCI, most cognitive training programs to date have also been conducted in group sessions [104]. However, most of our interviewed patients clearly stated that they would prefer to train individually at home or with the support of a care professional or partner. Therefore, it might be worthwhile to put more effort into designing and developing exergames that can be used individually at home. This would possibly also reduce the barriers of patients with mNCD to engage in exergame-based training programs in the long term.

Regarding training demands, the experts recommended focusing on game complexity to ensure a challenging but feasible cognitive demand. Physical exercise intensity should be maintained at a light to moderate level. To allow individualization of the cognitive demand in training, two main aspects should be considered: (1) task type (ie, choice of exergames to individually focus on neurocognitive functioning) and (2) task demands. To allow individualization of task demands, the following factors should be varied based on the experts’ recommendations: (1) stability support (use of handrail with both hands, one hand, or no support), (2) stepping direction, (3) game choice and tasks included, (4) game duration, or (5) game speed. Previous studies applying exergame-based motor-cognitive training in older adults with mNCD or MCI have applied relatively effortful, high cognitive demands [92], low [97] to moderate [96-98] physical exercise intensities, or have not reported the physical [76,91-95,99,100] or cognitive [76,91,93-100] exercise load or training progression in a clearly reproducible way. This exemplifies the fact that the optimal cognitive load for motor-cognitive training remains unknown. To the best of our knowledge, there has only been 1 meta-analysis to date that compared the effects of training interventions on cognitive functioning in relation to different task complexities and found no difference between simple and complex cognitive games [105]. Therefore, further investigations are needed to identify the optimal cognitive training demands and optimize the monitoring and progression of training programs. For physical exercise intensity, the recommendations of the interviewed experts are in line with those of previous studies applying exergame-based motor-cognitive training in older adults with mNCD or MCI. This also matches the analysis of the moderating variables of the training parameters that influence the effectiveness of the interventions. Based on meta-analytic results from motor-cognitive training in older adults with mNCD, moderate physical training intensity [45] has been shown to be most effective in improving cognitive function. Finally, moderate physical exercise intensity would also match the preferences of the patients interviewed in this study.

### Implications for Research

Our findings serve as a basis for user modeling, determination of therapeutic needs, and definition of a set of requirements for the game design and development of novel exergame-based training concepts. To increase the probability that the resulting training will be deemed feasible in future clinical practice, these considerations should be integrated to guide the decision process for the most suitable exergame design and intervention components when developing novel exergames and exergame-based training concepts.

### Limitations

The outcomes of this qualitative study must be interpreted with some caution, considering the following limitations. First, none of the interviewed patients with mNCD belonged to the clinical subtypes of mild frontotemporal NCD or mNCD with Lewy bodies. Depending on the clinical subtypes and the associated clinical pictures of the patients, different findings may have emerged from patient interviews. However, a substantial fraction (ie, ≥60%) of mild or major NCD is attributable to Alzheimer disease, whereas mild vascular NCD is the second most common cause of NCD after Alzheimer disease; frontotemporal NCD only accounts for approximately 5% of cases [9]. Therefore, the included study population appeared to be representative of these clinical subtypes. Second, owing to difficulties in recruiting patients, those screened for MCI according to predefined criteria were recruited in addition to patients with a clinical diagnosis of mNCD, which increased the heterogeneity of the study population. By contrast, in our project, we aimed to develop an individualized exergame-based training concept not only to treat clinically diagnosed patients with mNCD but also to prevent progression to dementia in individuals at risk who might not have been diagnosed (yet). Third, owing to the COVID-19 pandemic, all focus group sessions were held as web-based meetings. Face-to-face focus group sessions might have promoted livelier exchanges and may have led to additional insights.

### Conclusions

The psychosocial consequences of patients’ self-perceived cognitive deterioration may be more burdensome than the cognitive changes themselves. Older adults with mNCD prefer integrative forms of training (such as exergaming) and are primarily motivated by enjoyment or fun in exercising and the effectiveness of the training. Putting the synthesized perspectives of training goals, settings, and requirements for exergames and training components into context, our considerations point to opportunities for improvement in research and rehabilitation, either by adapting existing exergames to patients with mNCD or by developing novel exergames and exergame-based training concepts specifically tailored to meet patient requirements and needs.

## Appendix

Multimedia Appendix 1 Interview guides.

## Abbreviations

ADL: activities of daily living
CCT: computerized cognitive training
DSM-5: Diagnostic and Statistical Manual of Mental Disorders 5th Edition
HOA: healthy older adults
MCI: mild cognitive impairment
MIDE: Multidisciplinary Iterative Design of Exergames
mNCD: mild neurocognitive disorder
MTT: medical training therapy
NCD: neurocognitive disorders
PT: physical therapy

## Data Availability

The data supporting the findings of this study are available from the corresponding author (PM) on reasonable request.
