# Supplementary material for: Design Considerations for an Exergame-Based Training Intervention for Older Adults With Mild Neurocognitive Disorder: Qualitative Study Including Focus Groups With Experts and Health Care Professionals and Individual Semistructured In-depth Patient Interviews
Source: JMIR Serious Games. 2023 Jan 5;11:e37616. doi: 10.2196/37616 (PMC9853342; doi:10.2196/37616)
Supplement: Multimedia Appendix 1 [file games_v11i1e37616_app1.docx]

Supplementary File 1 – Interview Guides

| Table 1: Interview guide including topics and key questions to be discussed during the expert focus groups.  Abbreviations: mNCD, mild neurocognitive disorder; MCI, mild cognitive impairment | |
| --- | --- |
| Topic | Key Questions |
| (T1) Capabilities of MCI-Patients | What are the most prominent impairments of patients with mNCD/MCI (e.g. physical, cognitive, emotional)?  How do these impairments affect the usability of exergames / the “Senso” specifically? |
| (T2) Training Goals and Outcomes | What are the most prominent training goals of patients with mNCD/MCI?  In general, which training goals / capabilities (i.e. cognitive, physical) of patients with mNCD/MCI should be focused on based on your experience? |
| (T3) Treatment Experiences and  Preferences | What are previous experiences of patients with mNCD/MCI regarding different training- or rehabilitation concepts?  Where do patients with mNCD/MCI prefer to train (e.g. clinic, at home)? Do they need guidance? Are patients with mNCD/MCI capable of performing a home-based exergame training?  Would patients with mNCD/MCI be willing to wear a heart-rate sensor during training sessions? |
| (T4) Motivators for Training | What are reasons of patients with mNCD/MCI to carry on or quit exergame interventions?  Which existing „Senso“ games do patients with mNCD/MCI prefer? Why?  What elements should be included into future game- and interventions design to motivate patients with mNCD/MCI? |
| (T5) Exergame and Training Characteristics | What exergame- and intervention characteristics need to be considered for patients with mNCD/MCI specifically?  “Senso”-specific: Which of the existing games of the „Senso“ work well with patients with mNCD/MCI – which may be problematic? How could the existing game designs be adapted for patients with mNCD/MCI?  What novel game designs or -elements could be developed to optimally address the needs of patients with mNCD/MCI?  Based on your previous experience and knowledge: What would be the optimal exercise characteristics to maximize training effects regarding:   - - Exergame frequency (i.e. sessions per week)   - Exergame intensity/complexity   - Type of exergames   - Session duration [min]   - Duration of intervention [weeks]   - Exergame volume [min/week]   - Progression and periodization   - Variability   - Specificity (i.e. content of exergames) |

| Table 2: Interview guide including topics and key questions to be discussed during semi-structured patient interviews. | |  |
| --- | --- | --- |
| Topic | Key Questions | |
| (T1) Capabilities of MCI-Patients | How would you describe your ability to perform activities of daily living?  How would you describe your physical fitness?  How would you describe your mental fitness?  Can you operate a TV independently?  What do you feel most limited by in your everyday life? | |
| (T2) Training Goals and Outcomes | Which skills would you like to improve in order to increase your quality of life? | |
| (T3) Treatment Experiences and  Preferences | Have you already had experience with therapy options to improve the skills mentioned above?  If yes: How long have you used these options? What did you like about them? What do you think could be improved? In your opinion, what would be an optimal program to improve the aforementioned skills?  Have you already had experience with new technologies (for example, exergames)?  Would you be willing to try this form of training (exergames)?  Where would you like to train? In a center, at home, alone, with others?  Could you imagine training on your own?  Would you be willing to wear a heart rate monitor during training? | |
| (T4) Motivators for Training | What do you think could motivate you to train regularly?  What do you enjoy about training? | |
| (T5) Exergame and Training Characteristics | What requirements must be met by the training device so that you can use it independently?  What should be taken into account when planning the exergames and the training concept?  How often (number of training sessions per week) would you prefer to train with our exergame device?  How much time would you like to invest in your training per week with our exergame device?  At what intensity would you be willing to train with our exergame device?  How long should a training session with our exergame device last?  Over what period (in weeks) would you like to train with our exergame device?  Would you like a training program that is specifically tailored to your abilities?  How many exergames should each training session contain?  Do you have a wish or an idea what the games should contain? Are there any games you like to play? | |
